# A promising impact of oral administration of noscapine against imiquimod-induced psoriasis-like skin lesions

**DOI:** 10.22038/AJP.2023.21828

**Published:** 2023

**Authors:** Fahimeh Nourbakhsh, Seyed Hadi Mousavi, Pouria Rahmanian-Devin, Vafa Baradaran Rahimi, Hassan Rakhshandeh, Vahid Reza Askari

**Affiliations:** 1 *Medical Toxicology Research Center,* *Faculty of Medicine, Mashhad University* *of Medical Sciences, Mashhad, Iran*; 2 *Pharmacological Research Center of Medicinal Plants, Mashhad University of Medical Sciences, Mashhad, Iran*; 3 *Department of Cardiovascular Diseases, Faculty of Medicine, Mashhad University of Medical Sciences, Mashhad, Iran*; 4 *Applied Biomedical Research Center, Mashhad University of Medical Sciences, Mashhad, Iran*; 5 *International UNESCO Center for Health-Related Basic Sciences and Human Nutrition, Mashhad University of Medical Sciences, Mashhad, Iran*

**Keywords:** Psoriasis, Imiquimod, Inflammation, Noscapine, Methotrexate

## Abstract

**Objective:**

Psoriasis is a chronic inflammatory autoimmune disease. The effectiveness of noscapine has been employed as a helpful treatment for various disorders and subjected to recent theoretical breakthroughs.

**Materials and Methods:**

Psoriasis-like lesions were induced by topical application of 5% imiquimod (IMQ) (10 mg/cm^2^ of skin) in male *Balb/c* mice and then medicated with a single oral dose of methotrexate (MET) as a positive control or daily oral treatment of noscapine (5, 15 and 45 mg/kg). In this way, skin inflammation intensity, psoriatic itchiness, psoriasis area severity index (PASI) score, ear length, thickness, and organ weight were daily measured. At the end of the study, histological and immunohistochemical and enzyme-linked immunosorbent assays (ELISA, for pro-/anti-inflammatory factors) were performed in each ear.

**Results:**

IMQ caused psoriasis-like lesions. Noscapine markedly alleviated macroscopic parameters, namely ear thickness, ear length, skin inflammation, itching, and organ weight, as well as microscopic parameters including, pathology and Ki67 and p53, and tissue immunological mediators, such as tumour necrosis factor (TNF-α), interleukin (IL)-10, transforming growth factor (TGF-β), interferon-γ (IFN-γ), IL-6, IL-17, and IL-23p19 in the psoriatic skin in a concentration manner (p<0.05-<0.001).

**Conclusion:**

Therefore, noscapine with good pharmacological properties has considerable effects on psoriasis inflammation.

## Introduction

Psoriasis is a non-communicable, immune-mediated inflammatory skin disorder that disrupts the life-cycle of skin cells. Psoriasis causes thick, itchy, or red patches with silver manifestation, sometimes painful, as cells build up on the skin's surface. These patches mostly appear around the scalp, elbows and knees (Michalek et al., 2017; Parisi et al., 2013). For some people, psoriasis is just as annoying, but for others, it can cause disability, mainly if it is associated with arthritis. In addition, co-occurring psoriatic illnesses (metabolic syndrome, Crohn's disease, depression, cancer, and cardiovascular disease) can increase psoriasis's physical and psychological symptoms (Harden et al., 2015; Wu et al., 2012). The essential features of psoriasis are disorders associated with the skin that increase proliferation, hyperplasia and thickening of the epidermis (Rendon and Schakel, 2019). This process increases the risk of psoriatic lesions by increasing inflammatory mediators, mainly activated T cells and the recruitment of T cells in the epidermis. Psoriasis pathogenesis is complicated and dynamic, including specific epidermal Keratinocytes (KCs) and resident or recruited immune cells in the skin (Deng et al., 2016; Di Meglio et al., 2014). 

It was originally thought that the vital factor of the illness was initiated by KC hyper-proliferation coupled with aberrant epidermal division. Cytokines and chemokines are generated in the skin with indigenous cells like KCs, Langerhans cells (LCs), mast cells, and infiltrating cells such as lymphocytes and neutrophils. 

At the start of different skin disorders, it is well understood that KCs encourage an amplification of the inflammatory response by increasing the production of tumour necrosis factor TNF-α and interferon (IFN)-γ. These are primary inflammatory factors since they stimulate secondary inflammatory cytokines and chemokine production (Chiricozzi et al., 2018). Evidence suggests that T-helper 17 (Th17)-compatible immune systems play an important role in the induction and severity of psoriasis. Moreover, interleukin-23 (IL-23) acts as a critical cytokine for the severity of inflammation in psoriasis. In addition, Th17 cells, primarily type A, IL-22 and TNF-α play an influential role in keratinocyte proliferation. According to previous research, CD4^+^ and CD8^+^ T-cells play a critical role in the pathogenesis of psoriasis, both starting and maintaining inflammation.

On the other hand, T-Cells have been shown to play an essential role in psoriasis. Although the specific aetiology of this disease is unknown, there is a preliminary document that interleukin (IL)-23/IL-17A has a considerable influence on the disease's development. Several proinflammatory mediators such as IL-23, IL-17A/F, IL-20, IL-22, IL-6, and TNF-α promote disease progression (Furue et al., 2020). Numerous methods have been performed to investigate and induce psoriasis, especially in animal models. Using imiquimod (IMQ) is one of the best methods to regulate the immune response in the induction of psoriasis. It has been demonstrated that IMQ may be used to cure active lesions and keratosis by using dendritic cells, particularly plasma dendritic cells (PDCS), Natural Killer (NK) cells, and T lymphocytes that generate IFN and IL-17. In addition, Imiquimod boosts the innate immune system by stimulating the toll-like receptor 7/8 (TLR-7/8), which is often implicated in pathogen detection (Conrad and Gilliet, 2018; Hawkes et al., 2018). 

Methotrexate is used weekly as a common medication for mild to moderate patients who have psoriasis. Despite the wide range of methotrexate treatments, numerous side effects have been reported, such as internal bleeding, gastrointestinal disorders, drowsiness, nausea, and vomiting in users of this drug (Bischoff et al., 1971; Chan and Cronstein, 2010). Therefore, introducing new medications with the most negligible adverse effects and disease control and relief is a particular priority (Farhangian and Feldman, 2015; Roenigk et al., 1988). In particular, these therapies focus on preventing further disease complications, disabling them, and improving clinical symptoms. The most crucial goal in treating and preventing recurrences of lesions in patients with psoriasis is to control the intensity of cell growth with the help of topical, oral or injectable therapies. The severity and type of the disease are very effective in choosing treatment. Common treatments for psoriasis include corticosteroids, vitamin D3 analogues, and calcineurin inhibitors. Common topical treatments include phototherapy, and other standard treatments include methotrexate, cyclosporine, and acitretin (Dubois Declercq and Pouliot, 2013; Sean A Sukal et al., 2006). Noscapine or narcotin is a benzylisoquinoline alkaloid found in dark poppy plants and is safe (Nourbakhsh and Askari, 2021; Rahmanian-Devin et al., 2021a). Numerous studies have shown that noscapine extensively prevents cell proliferation in a wide range of cancer cells, especially drug-resistant strains (Chen et al., 2015; Dahlström et al., 1982). In addition, numerous studies have reported the positive effects of noscapine on humoral immunity, stroke and cough. It functions as an antitussive, antineoplastic agent, an activator of cell death, and a plant metabolite (Chen et al., 2015; Nourbakhsh and Askari, 2021; Rahmanian-Devin et al., 2021a). Hence, in the current experiment, we examined the impact of oral noscapine in the animal model of IMQ-induced psoriasis.

## Materials and Methods

**Chemicals and reagents**

Noscapine powder (purity >99.5%) was from FaranShimi® Company, Hamedan, Iran. Cold cream was also a standard material and prepared by the Farabi® Company in Tehran, Iran. All chemicals and reagents were obtained from Sigma-Aldrich except as otherwise specified (St. Louis, MO, USA). Furthermore, 5% imiquimod propionate cream patches (Aldara) were provided from MEDA, Sweden. The cytokines kits were bought from the e-bioscience company (St. Louis, MO, USA). Immunohistochemical (IHC) examination kits to detect Ki-67 and p53 proteins were from GenomeMe (Cat # IHC053) and ZYTOMED, (Cat # BRB040), respectively.

**Ethical reiteration**

In this research, male inbred *Balb/c* mice (n=42) take from the Faculty of Medicine animal laboratory, Mashhad University of Medical Sciences, Mashhad, Iran. All the mice were randomly separated into six groups (n=7/group, weighing 25.2±4.6 gr). All animal handlings were performed following the Mashhad University of Medical Sciences ethical committee authorised all animals' operations by animal experimentation and treatment rules (2020–Sep–07, IR.MUMS.MEDICAL.REC.1399.463).

**Psoriasis induction and trial procedure**

**Figure 1:**
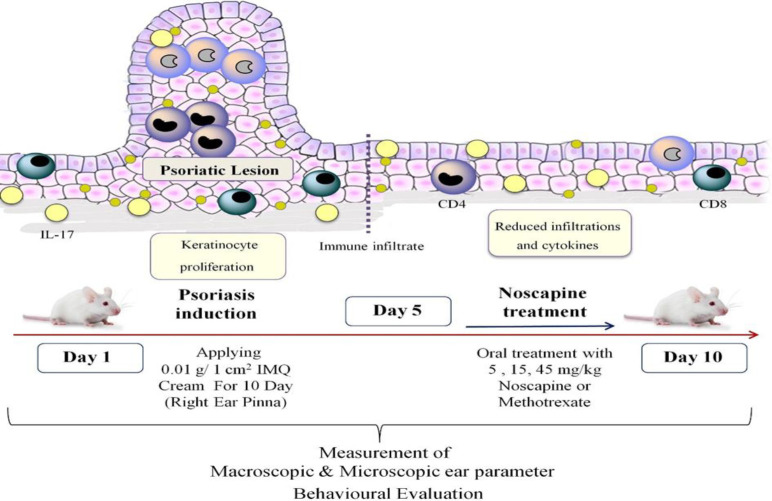
Noscapine alleviates IMQ-induced psoriasis-like skin lesions.

Psoriasis was induced from day one using IMQ; a certain amount of this cream (1 cm^2^ of skin; 0.01 gr of IMQ cream) was applied to the right ear of mice in all groups. To examine the process of disease induction as well as scoring based on psoriasis area severity index (PASI) scores, all groups were photographed daily according to the previous reports (Na Takuathung et al., 2018; Nourbakhsh et al., 2022). Grouping was done as follows, and the procedures are illustrated and detailed in Figure 1 and Table 1, respectively. 

Group I (sham group): The first group received a daily topical cold cream on the right ear pinna as the vehicle; Group II (negative control group): The group received a daily topical dosage of 5% IMQ cream (0.01 gr IMQ cream in 1 cm^2^ of skin) on the right ear pinna. Additionally, after 30 min the mice received daily topical cold cream; Group III (positive control group): The group received a daily topical dosage of 5% IMQ cream on the right ear pinna, and then received a single dose of oral methotrexate (MET) on the fifth day; Group IV-VI (treatment groups): The group received a daily topical dosage of 5% IMQ cream on the right ear pinna and received 5, 15 or 45 mg/kg/d noscapine.

Based on the preliminary investigation, all doses were estimated using a three-fold increase in dosage. Figure 1 depicts an illustration of the experimental techniques of mice showing the severity of redness, inflammation and other macroscopic factors.

**Table 1:** An illustration of the experimental procedures

**Treatment day**		**Groups**	**Protocol**
Day 1-10	Daily topical cold cream (10 mg/cm^2^ of skin)	Sham	(n=7/each group) Psoriatic lesion & Noscapine therapy
	Daily topical dosage of 5% IMQ cream (10 mg/cm^2^ of skin)	Negative Control	
	Daily topical dosage of 5% IMQ + single dose oral methotrexate (MET)	Positive Control	
	Daily topical dosage of 5% IMQ + orally received 5 mg/kg noscapine	Treatment	
	Daily topical dosage of 5% IMQ + orally received 15 mg/kg noscapine		
	Daily topical dosage of 5% IMQ + orally received 45 mg/kg noscapine		

**Inflammation scoring**

The levels of PASI were calculated using erythema levels (Image J software was used), thickness, and scaling on the affected ear pinna surface (Na Takuathung et al., 2018; Nourbakhsh et al., 2022). Moreover, ear thickness and length were also assessed daily utilising digital callipers (BEC, China). These are the parameters for the quantity of epidermal development and inflammation, determined by the increase in ear thickness and inflammation (Na Takuathung et al., 2018; Nourbakhsh et al., 2022). 

**Psoriatic itching scoring**

Mice were examined after being habituated to a Plexiglas recording arena twice for 60 minutes. In addition, mice were videotaped from above for 60 minutes after each noscapine treatment. A scratch bout was defined as one or more rapid back-and-forth hind paw motions aiming towards and contacting the treated region, resulting in toe licking, biting, or planting of the hind paw on the floor. Grooming actions and rear foot motion away from the treatment area (e.g., ear scratching) were not recorded. The cervical region of the spinal cord (C3–C4) was also chosen for H&E staining and investigation of cellular infiltrations (Na Takuathung et al., 2018; Nourbakhsh et al., 2022; Sakai et al., 2016). 

**Evaluating the spleen, ear, and body weight of mice**

Animals were sacrificed at the end of the research using in depth anaesthesia using ketamine (100 mg/kg) + xylazine (10mg/kg) and acepromazine (3mg/kg) and then incubated with CO_2_ chamber for 2 min. After that, tissues were dissected and stored at -70°C. Each mouse's spleen and ear were separated in this manner, weighed, photographed, and placed in a freezer at -70°C or 10% v/v formalin for subsequent assessments.

**Histopathological and immunohistochemical evaluation**

The tissue sample was taken from the right ear pinnas and treated in 10% v/v neutral-buffered formalin. Each sample was put in a Falcon tube with 10% formalin and individually transported to the pathology laboratory. The pieces were soaked in paraffin blocks before being sliced to a 4 μm thickness. The stained tissues were photographed with a digital camera microscope (Nikon, Eclipse E200). According to the previous reports, acanthosis, parakeratosis and thickening of the subepidermal layer were among distinct groups' pathologic alterations in the ear pinna (Na Takuathung et al., 2018; Nourbakhsh et al., 2022; Sakai et al., 2016). 

This technique was performed to detect Ki-67 as a marker of cell proliferation and p53 as a marker of cell apoptosis based on the kit protocols. These variables were measured on a 0–10 scale (0=no change from untreated ears; 10=maximum) (Na Takuathung et al., 2018; Nourbakhsh et al., 2022; Sakai et al., 2016).

**Assay of cytokines production**

The Bradford protein measurement method was utilised to determine the whole protein level in each sample accordingly (Askari et al., 2020; Askari et al., 2019; Baradaran Rahimi et al., 2020; Bradford, 1976; Rahmanian-Devin et al., 2021b). Furthermore, following the euthanisation, the ear pinnas of the mice were excised and kept at -70^o^C to evaluate cytokine (TNF-α, TGF-β, IFN-γ, IL-10, IL-6, IL-17 and IL-23p19) measurement in skin tissue, based on the previous studies' results. Finally, ELISA was performed following the manufacturer's instructions based on the kit protocols (Askari et al., 2020; Askari et al., 2019; Baradaran Rahimi et al., 2020; Rahmanian-Devin et al., 2021b) 

**Analysis of data**

Data were analysed using GraphPad Prism ® software version 8 (San Diego, CA) and expressed as means±SD or median depending on whether they were parametric or non-parametric. A two-way analysis of variance (ANOVA) was used to compare groups with Sidak's multiple comparisons *post hoc test*. For clinical ratings, repeated measures two-way ANOVA was used with the Dunnett-T3 multiple comparisons *posthoc test*. The Kruskal-Wallis test and Dunn's *posthoc* multiple comparisons test were used to analyse non-parametric data. If the SD was not equal, the Brown-Forsythe test was used, and a statistical test for group variance equality was based on an analysis of variance. P values p≤0.05, ≤0.01 and ≤0.001 were judged statistically significant. 

**Table 2:** The summarised protocols of the present study

Two-way ANOVA Dunnet	F (50, 360) = 50.1	501.0	F (5, 36) = 241.1	F (10, 60) = 131.5 p<0.0001, N=11	Thickness
Two-way ANOVA Sidak	F (60, 420) = 16.73	11	F (6, 42) = 128.0	F (10, 60) = 375.3 p<0.0001, N=11	Erythema
Two-way ANOVA Sidak	F (60, 420) = 16.73	11	F (6, 42) = 128	F(10,60)=375.3 p<0.0001, N=11	Scales
Two-way ANOVA Sidak	F (50, 360) = 86.43	11	F (6, 42) = 589.1	F(10,60)=722.1 p<0.0001, N=11	Total Score
Two-way ANOVA Dunnet	F (60, 360) = 76.99	11	F (5, 36) = 334.5	F(10,60)=440.0 p<0.0001, N=11	Ear Length
Two-way ANOVA Dunnet	F (60, 360) = 16.73	11	F (6, 42) = 128	F(10,420)=375.3 p<0.0001, N=11	Scratch bouts
One-way ANOVA Dunnet	F (6, 36) = 5.24	7	7	F (2.70,18.32) = 15.12 p<0.0001	Body Weight
One-way ANOVA Dunnet	-	7	1	F (6, 42) =17.32 p<0.0001	Spleen Weight
One-way ANOVA Dunnet	-	7	1	F (6, 42) =11.78 p<0.0001	Ear index
One-way ANOVA Dunnet	-	7	1	F (6, 36) = 29.7 p<0.0001	Spleen index
Brown Forsythe test Dunnet T3	-	35	7	F (6, 22) = 59.76 p<0.0001	TNF-ơ
Brown Forsythe test Dunnet T3	-	35	7	F (6, 22) = 53.7 p<0.0001	IL-10
Brown Forsythe test Dunnet T3	-	35	7	F (6, 22) = 61.78 p<0.0001	TGF-β
Brown Forsythe test Dunnet T3	-	35	7	F (6, 22) = 42.65 p<0.0001	IL-6
Brown Forsythe test Dunnet T3	-	35	7	F (6, 22) = 62.7 p<0.0001	IFN-ɣ
Brown Forsythe test Dunnet T3	-	35	7	F (6, 15) = 38.4 p<0.0001	IFN-ɣ/IL-10
Brown Forsythe test Dunnet T3	-	35	7	F (6, 15) = 63.74 p<0.0001	IL-17A
Brown Forsythe test Dunnet T3	-	35	7	F (6, 22) = 63.4 p<0.0001	IL-23p19

The data and statistical analysis followed the preclinical pharmacology standards for experimental design, analysis, data sharing, and presentation (Alexander et al., 2018; Curtis et al., 2015; George et al., 2017). The statistical analysis was summarised in Table 2.

## Results

**Psoriasis induction**

**Figure 2:**
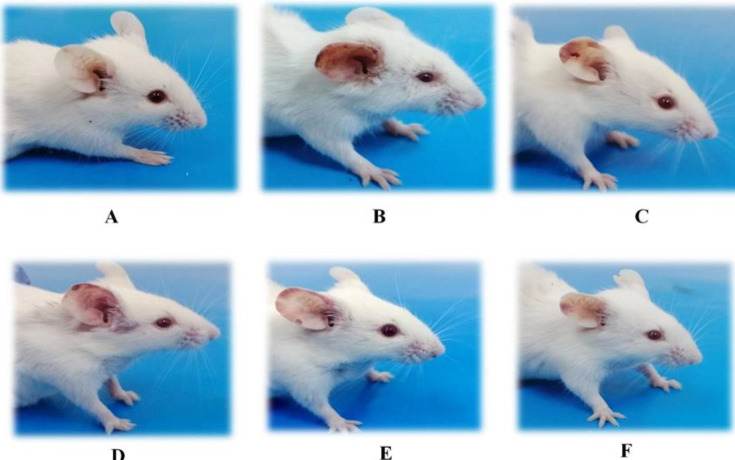
The severity of psoriasis-like symptoms in all groups for ten days was showing A: sham group, B: negative control group, C: positive group, D: sample group received IMQ plus 5 mg/kg noscapine, E: sample group received IMQ plus 15 mg/kg noscapine, F: sample group received IMQ plus 45 mg/kg noscapine.

All of the mice's features and health conditions were normal throughout the research, involving food and water intakes, behavioural indicators, respiratory patterns, and cardiovascular measurements. Two or three days after starting IMQ treatment, the mice's right ear pinna displayed erythema, scaling, and thickening signs. However, until the IMQ treatment was finished, the severity of psoriasis-like symptoms in mice group II gradually improved (until day 10). On the other hand, the mice in group I, who were administered cold cream, exhibited no signs of inflammation on their right ear pinna. Similarly, other groups given different doses of noscapine were photographed every day and scored (Figure 2). 

**Noscapine's effects on the ear thickness, erythema, scales and ear length**

**Figure 3:**
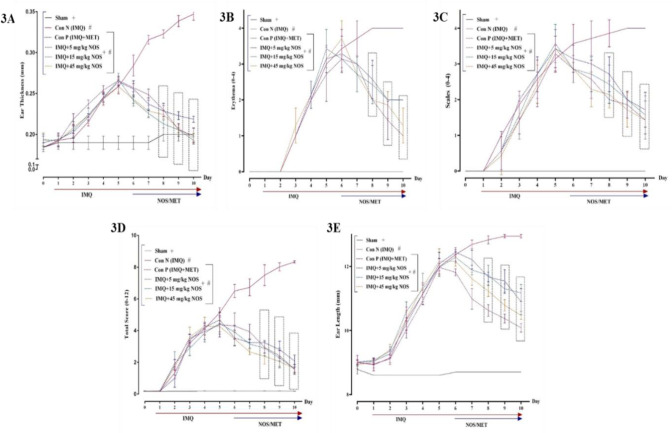
Phenotypical observations of ear pinna in treated mice groups. The symptoms of the thickness (A), erythema (B), scaling (C), the total score (D) and ear length (E) after commencing IMQ administration right ear pinna of the mice. The results are expressed as the mean±SD (n=7 in each group). The results indicate significant changes in the sham and negative control groups. # compared to the sham group (#p<0.001). + compared to the negative control group (+p<0.001). A two-way analysis of variance (ANOVA) was used to compare groups, followed by Sidak's multiple comparisons *post hoc test*. For clinical ratings, repeated measures two-way ANOVA was used with the Dunnett Dunnett-T3 multiple comparisons *posthoc test*. The Kruskal-Wallis test and Dunn's *posthoc* multiple comparisons test were used to analyse non-parametric data. If the SD was not equal, the Brown-Forsythe test was used, and a statistical test for group variance equality was based on an analysis of variance.

In this way, the PASI scores peaked on day five after IMQ exposure, showing that psoriasis-like dermatitis had been effectively produced in the IMQ-receiver mice. Furthermore, according to the results, the right ear pinna of the mice showed symptoms of erythema, scaling and thickening after administration of IMQ (Figure 1). In addition, the results indicated that the IMQ 5% application considerably increased the ear thickness compared to the sham group (p<0.001, Figure 3A). In this regard, the tendency of thickness decreases on day seven in all dosages (noscapine 5, 15 and 45 mg/kg, p<0.001) and on day six in noscapine 45 mg/kg (means±SD: 0.229±0.002, p<0.001). 

On day five of psoriasis induction, the data revealed an enhancement in inflammation and redness in all groups. According to the present study's findings, all dosages of noscapine significantly reduced the amount of erythema generated by the IMQ application (p<0.001 for all cases, Figure 3B). It also confirmed that treatment with methotrexate (means±SD: 3.10±0.0, p<0.001) and noscapine 45 mg/kg (means±SD: 2.71±0.48, p<0.001) significantly reduced redness from day 7 in the treatment group. Different groups' scales (0-4) were revealed to represent the focused production of inflammatory and conspicuous plaques caused by skin epithelial cell expansion. Each mouse earns a score ranging from 0 to 4 depending on erythema, scaling, and thickness (Figure 3C). It indicated that the application of different concentrations of noscapine (5, 15, 45 mg/kg) significantly reduced the scale range from day seven compared to the control group (p<0.001 for all cases, Figure 3C). In this regarding treatment with different concentrations of noscapine on day seven of therapeutic dosages of noscapine 15 mg/kg (means±SD: 2.71±0.48, p<0.001) and noscapine (means±SD: 2.28±0.48, p<0.001) had a significant influence on the treatment of drug-receiving groups. In addition, our results confirmed that the total score (erythema plus scaling plus thickness) in the control group received IMQ was significantly increased in comparison to the sham group (p<0.001, Figure 3D). Additionally, the noscapine administration (5, 15, 45 mg/kg) and also methotrexate considerably diminished the total score from day seven compared to the sham group (p<0.001 for all cases, Figure 3D). The results showed a considerable increase in the level of ear length during the use of IMQ compared to the sham group (p<0.001, Figure 3E). According to the results of this study, the ear length of mice increased according to the topical administration of IMQ compared to the sham group (p<0.001, Figure 3E). In contrast, the application of different concentrations of noscapine (5, 15, 45 mg/kg) significantly attenuated the ear length from day eight compared to the control group (p<0.001 for all cases, Figure 3E). In contrast, oral administration of noscapine, especially doses 15 mg/kg (means±SD: 11.72±0.30, p<0.001) and 45 mg/kg (means±SD: 11.22±0.17, p<0.001) from day 8 of treatment, has been effective in reducing inflammation and redness and thus reducing the length of the mouse ear (Figure 3E).

**Noscapine's impact on the spleen and body weight**

**Figure 4:**
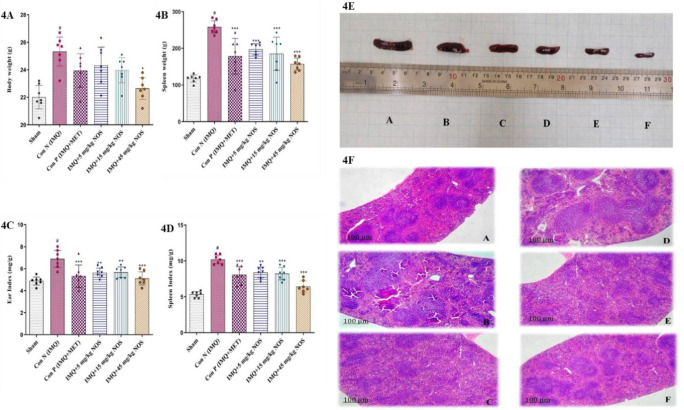
The investigation of mice in terms of body weight (A), the weight of the spleen (B), ear index (C), the spleen index (D), representative photos of the spleen (E) and pathological investigation of the spleen (F) respectively showed in fig 4A-F. Figure 4E illustrates the spleen length of each group, including A: negative control group, B: positive control group, and C: positive control group. D: IMQ plus 5 mg/kg noscapine was given to the sample group, E: IMQ plus 15 mg/kg noscapine was given to the sample group, and F: IMQ plus 45 mg/kg noscapine was given to the sample group. The results are expressed as the mean±SD (n=7 in each group). The results indicate significant changes compared to the negative control group. *compared to the negative control group (***p< 0.001, **p< 0.01 and *p< 0.05). # compared to the sham group (#p<0.001). For clinical ratings, repeated measures two-way ANOVA was used with the Dunnett Dunnett-T3 multiple comparisons posthoc test. The Kruskal-Wallis test and Dunn's posthoc multiple comparisons test were used to analyse non-parametric data. If the SD was not equal, the Brown-Forsythe test was used, and a statistical test for group variance equality was based on an analysis of variance.

In comparison to the sham group, IMQ 5% w/w on the ear resulted in a substantial rise in body weight (p<0.001, Figure 4A). However, when compared to the sham group, diverse doses of noscapine (5, 15, 45 mg/kg) significantly and concentration-dependently decreased the high body weight (p<0.001 to 0.05 in all cases, Figure 3A). Furthermore, the daily investigations indicated a substantial weight reduction in the groups that received 45 mg/kg noscapine (means±SD: 22.64±0.32, p<0.05) compared to the negative control group when assessing mice's body weight. Additionally, IMQ 5% w/w on the ear resulted in a significant elevation in the spleen weight compared to the sham group (p<0.001, Figure 4B). It was indicated that methotrexate or noscapine treatment (p<0.001, for all cases, Figure 4B) notably reduced the elevated spleen weight compared to the sham group (Figure 4B). The indexes for the ear (Figure 4C) and the spleen (Figure 4D) were determined by dividing the total weight of each organ by the body weight. As a consequence, the IMQ-control group had higher ear (p<0.001, Figure 4C) and spleen weight (p<0.001, Figure 4D) indices than the sham group. When compared to the sham group, using each methotrexate or noscapine (5, 15, 45 mg/kg) significantly reduced the raised ear (p<0.001-0.01 for all instances, Figure 4C) and spleen (p<0.001-0.01 for all cases, Figure 4D) indices. 

The parameters of the ear index in the groups receiving 45 mg/kg noscapine (means±SD: 5.11±0.24, p<0.001) compared to the negative control group were particularly significant. In addition, the parameters of the spleen index in the groups receiving 15 mg/kg noscapine (means±SD: 8.24±0.35, p<0.001) and 45 mg/kg noscapine (means±SD: 6.42±0.31, p<0.001) compared to the negative control group showed remarkable results. After measuring and weighing the spleen tissue in each group, spleen samples were photographed. Hence, Figure 4E illustrates the spleen length of each group. The abnormal red pulps of the spleen in the negative control group and non-spleen tissue destruction in treated specimens are shown in images from pathological examinations (Figure 4F). The findings showed that spleen white pulp reduction was dose-dependent in various groups. 

**Noscapine's effect on scratching behaviour**

**Figure 5:**
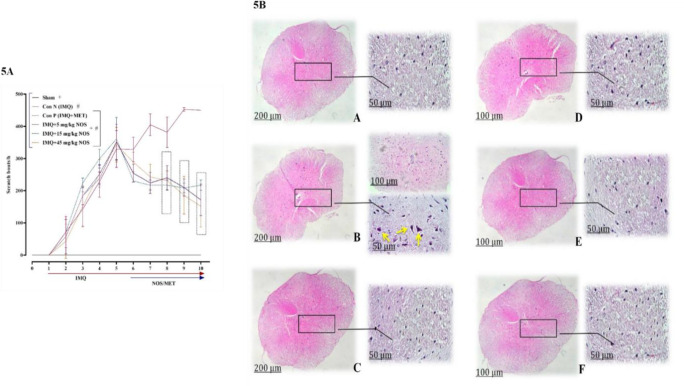
The number of scratching behaviour in IMQ-induced psoriasis-like skin (A) and also H&E staining of cord sections revealed marked areas of necrosis with vacuolisation of cells (B). The results are expressed as the mean±SD (n=7 in each group). The results indicate significant changes compared to the negative control group. * compared to the negative control group (^***^p<0.001,^ **^p<0.01 and ^*^p<0.05). # compared to the sham group (#p<0.001). A two-way analysis of variance (ANOVA) was used to compare groups, followed by Sidak's multiple comparisons *post hoc test*.

Because scratching is familiar in patients with psoriasis, we looked at the number of spontaneous scratching in mice ear pinna receiving the noscapine compared to the sham group (p<0.001, Figure 5A). In one hour, the number of itching times of the group receiving IMQ reached its maximum on day five compared to the sham group (means±SD: 329.42±57.11, p<0.001). 

Additionally, from day seven, a decrease in scratching was considerably observed in groups that received 15 mg/kg noscapine (means±SD: 242.85±39.0, p<0.001) and 45 mg/kg noscapine (means±SD: 217.14±16.03, p<0.001). For pathological examinations, H&E staining of spinal cord sections indicated marked areas of necrosis with vacuolisation of cells (p<0.001 for all cases, Figure 5B).

**Noscapine's effect on the concentration of the cytokine**

**Figure 6:**
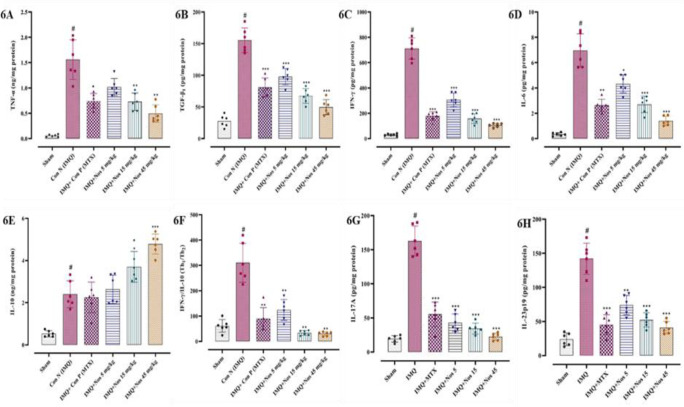
The concentration of cytokines levels such as TNF-α (A), TGF-β (B), IFN-γ (C), IL-6 (D), IFN-γ /IL-10 (E), IL-10 (F), IL-17A (G) and IL-23p19 (H) respectively showed in Figure 6A-H. The results are expressed as the mean±SD (n=7 in each group). The results indicate significant changes compared to the negative control group. * compared to the negative control group (^***^p<0.001,^ **^p<0.01 and ^*^p<0.05). # compared to the sham group (#p<0.001). The Kruskal-Wallis test and Dunn's *posthoc* multiple comparisons test were used to analyse non-parametric data. If the SD was not equal, the Brown-Forsythe test was used, and a statistical test for group variance equality was based on an analysis of variance.

Inflammatory cytokines, which are necessary for inflammation, were measured in right ear pinna of sham, IMQ-treated, and noscapine-treated mice using ELISA assays. TNF-mediated cytokines, such as TGF-β, IL-6, IFN-γ, IL-17, and IL-23p19, were significantly higher in-ear pinna samples from IMQ-treated mice than in the sham group (p<0.001 for all cases, Figure 6A-H). In contrast, the IMQ-treated mice and the noscapine-treated animals demonstrated a substantial dose-dependent drop in TNF, TGF, IFN-γ, IL-6, IFN-γ /IL-10, IL-17A, and IL-23p19, as well as elevations in IL-10 expression. The results showed that the IMQ-received group alone has an excessive increase in TNF-α level (means±SD: 1.56±0.35, p<0.001, Figure 6A). Similarly, TGF-β level (means±SD: 155.4±17.74, p<0.001, Figure 6B), IFN-γ (means±SD: 712.16±76.57, p<0.001, Figure 6C), IL-6 (means±SD: 6.96±1.19, p<0.001, Figure 6D), IL-10 (means±SD: 2.40±0.58, p<0.001, Figure 6E), IFN-γ /IL-10 (means±SD: 310.73±70.35, p<0.001, Figure 6F) ratio also increased in the IMQ received mice. Similar results have been reported for IL-17A (means±SD: 162.66±20.01, p<0.001, Figure 6G) and IL-23p19 (means±SD: 142.16±20.73, p<0.001, Figure 6H) in this regard.

**Effect of noscapine on the histopathological investigation**

**Figure 7:**
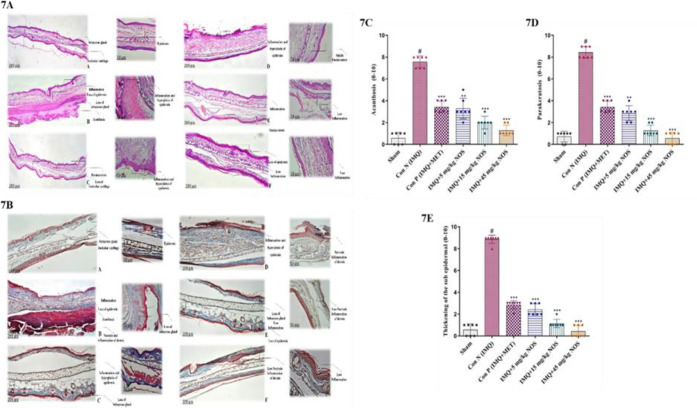
Hematoxylin and eosin (A) and trichrome staining (B) were presented for each sample. The pathologic changes in the ear pinna of various groups were included: acanthosis (C), parakeratosis (D) and thickening of the subepidermal layer (E). The data were expressed as the median±IQR (n=7 in each group). The results also indicate significant changes compared to the negative control group. * compared to the negative control group (^***^p<0.001,^ **^p<0.01 and ^*^p<0.05). # compared to the sham group (#p<0.001). The Kruskal-Wallis test and Dunn's *posthoc* multiple comparisons test were used to analyse non-parametric data.

The right ear pinna sections of IMQ-treated mice showed considerably improved acanthosis (p<0.001, Figure 7C), parakeratosis (p<0.001, Figure 7D) and thickening of the subepidermal layer (p<0.001, Figure 7E) in comparison to the sham group. The findings also showed that noscapine had a good effect on decreasing inflammation and tissue degradation at all therapeutic levels (Figures 7C, D, E).

Furthermore, when comparing the noscapine and methotrexate-treated mice to the IMQ-treated group, the thickening of the subepidermal layer was considerably reduced (p<0.001-0.01 for all cases, Figure 7E). As a result, in 15 and 45 mg/kg noscapine treatment, virtually mice recovered from IMQ-induced hyperplasia of the epidermal and subcutaneous tissue, and relatively moderate inflammatory responses were reported. The findings suggest that noscapine, particularly at the high treatment doses, could reduce inflammation and pathological scores in treated animals. Furthermore, the results indicated that noscapine, particularly at the levels tested, could reduce inflammation and pathological scores in treated animals. 

**Effect of noscapine on the immunohistochemical assessment**

**Figure 8:**
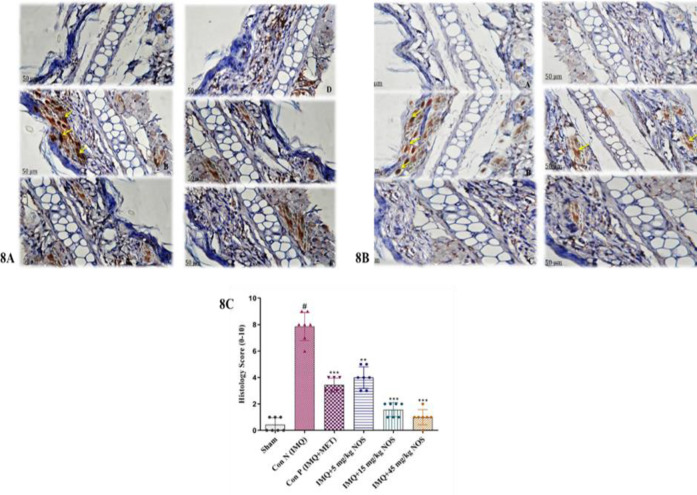
Immunohistochemistry (IHC) staining for p53 protein (A) and Ki-67 protein (B) as specific antigens in an ear tissue based on antigen-antibody reaction by light microscopy. The histopathological score of examined tissues (C) is expressed as the median±IQR (n=7 in each group). The results also indicate significant changes compared to the negative control group. * compared to the negative control group (^***^p<0.001,^ **^p<0.01 and ^*^p<0.05). # compared to the sham group (#p<0.001). The Kruskal-Wallis test and Dunn's *posthoc* multiple comparisons test were used to analyse non-parametric data.

Figure 8C showed a semi-quantitative histological score of the analysed tissues on a scale of 0–10. We discovered that the histology score in the IMQ-control group was considerably higher than in the sham group (p<0.001, Figure 8C). In contrast to the control group, administering noscapine (5, 15 and 45 mg/kg) or methotrexate substantially lowered the histology score (p<0.001 for all instances, Figure 8C).

## Discussion

The present study is the only study that we are aware of that assessed the protective benefits of noscapine against the IMQ-induced mice model. Consequently, we discovered that noscapine decreased the inflammation and psoriatic manifestation of IMQ by reducing ear thickness and length and altering the apoptosis (p53) and cell proliferative (Ki-67) factors in a concentration-dependent and substantial manner. Evidence is accumulating that noscapine has vigorous anti-oxidant activity and significantly reduces the inflammatory biomarker expression, leading to decreased cytokine production. 

As psoriasis is an immune-mediated inflammatory skin disorder, this extensive evidence of pharmacological activity may indicate the potential for using noscapine in treating psoriasis. The study stated that noscapine could reduce inflammation and thickness of psoriatic lesions in the IMQ-induced mouse model due to its high skin permeability. As shown in other similar studies, keratinocytes are the most common cells in the epidermis, and they help activate innate immunity by acting as a monitor against external infections (Benhadou et al., 2019). In a similar study, researchers found that rosmarinic acid had antiproliferative properties in the inflammatory Hacat cell line and anti-psoriasis potential in an IMQ-induced psoriasis-like mice model (Zhang et al., 2021). After oral medication, rosmarinic acid lowered the PASI score and reduced skin inflammation. Other similar studies have been performed in this field. Our findings align with this research that suggested noscapine like rosmarinic acid might be a promising anti-psoriasis treatment.

Therefore, in addition to examining the therapeutic effects of noscapine, the present study also examined the preventive and restorative effects of noscapine on pruritus. In addition to skin lesions, itching is another complaint of patients with psoriasis. This study shows that imiquimod-treated mice could be considered a good model for studying the neural processes driving itch and itch sensitisation in psoriasis. Psoriasis-like skin symptoms such as erythema, scales, epidermal thickness, and inflammatory cell infiltration are seen in imiquimod-treated mice, which is consistent with earlier research (Van Der Fits et al., 2009). Imiquimod-treated mice scratch more spontaneously, indicating that persistent itch is present in this paradigm. It was shown that imiquimod-treated mice develop alloknesis, which is a symptom of itch sensitivity seen in psoriasis patients. A similar study confirmed that histamine H_1_ receptor antagonists decreased spontaneous scratching only on day 2, not affecting alloknesis (Szepietowski et al., 2002). Similar studies look at the function of nonpeptidergic nerve fibres in a psoriasis model. Similarly, the role of neurturin in itching and intraepidermal fibre density in a mice model of psoriasis was demonstrated (Sakai et al., 2017). 

Therefore, in addition to examining the therapeutic effects of noscapine, the present study also examined the preventive and restorative effects of noscapine on scratching behaviour. In line with several studies to evaluate the extent of itching in patients with psoriasis, therapies focused on the molecular signalling pathway of scratching behaviour have therapeutic itching as a chronic inflammatory disease.

Psoriasis is involved in the proliferation of skin epithelial cells (Michalek et al., 2017). Methotrexate and clobetasol have been shown to play a role in cell proliferation and the immune system and can have promising therapeutic effects. Noscapine has key clinical benefits such as antitussive characteristics and cell proliferation as an ancient medication. As a result, we attempted to assess the efficacy of topical administration of noscapine in an animal model of psoriasis. Based on the findings of this study, studies in the human phase could be conducted. Furthermore, as an old drug, noscapine provides important therapeutic qualities such as antitussive properties and cell proliferation. As a result, we attempted to examine the efficacy of topical administration of noscapine in an animal model of psoriasis; therefore, further investigations in the human phase could be done based on the findings of this study. 

In the current study, Image J software was used to measure the thickness of the epidermis in psoriatic lesions. The analysis indicates that the decrease in thickness and skin irritation began on day 7 in all dosages and began on day 6 in the noscapine 45 mg/kg dose. The current study's results were consistent with earlier research findings indicating that topical capsaicin treatment considerably reduces skin inflammation. This decrease in thickness was not notable in the positive control group receiving MET beginning on day 6 of treatment, indicating that the oral version of noscapine outperformed the MET administration (Desai et al., 2013). A similar pattern of results was achieved in the therapeutic benefits of curcumin on lowering inflammation and skin thickness in psoriasis-induced rats starting on day 6 of treatment (Sun et al., 2013). Numerous studies have found that noscapine has a robust and considerable anti-oxidant action in lowering NF-kB expression, which leads to a decrease in cytokine production by T-cells (Ebrahimi, 2020; Sung et al., 2010). 

In fact, we discovered that noscapine could decrease fibrosis and inflammation in the ear by lowering inflammatory mediators (TNF-α, TGF-β, IFN-γ, IL-6, IL-17, and IL-23p19), raising anti-inflammatory cytokine IL-10, and modifying apoptosis (p53) and cell proliferation (Ki-67). Currently, we show that noscapine inhibited the generation of proinflammatory mediators via TNF-α /IFN-γ activation in this study. According to our findings, noscapine has high anti-oxidant activity and significantly reduces the transcription of the TNF-α signalling indicator, resulting in a decrease in T-cell cytokine output. TNF-mediated cytokines, TGF-α, IL-6, and IFN-γ, were significantly higher in mice in the positive control group. Our findings are consistent with prior research showing that noscapine has anti-inflammatory characteristics, mainly through modulation of the NF-γB signalling pathway in chemotherapeutic drugs (Tomar et al., 2017). Similar studies reveal that brominated noscapine analogues (Br- noscapine and Red-Br- noscapine) suppress TLR-mediated TNF-α and nitric oxide (NO) generation in human and murine macrophages with no sign of cellular injury. Brominated noscapine analogues also reduced cytokine/chemokine-induced sterile inflammation (a non-TLR ligand) (Tomar et al., 2017). Similar data indicate that rosmarinic acid reduces IL-23 production, suppresses Th17-dominated inflammation, and down-regulates the Jak2/Stat3 signal pathway, suppressing psoriasis-like skin inflammation in vivo and in vitro. Accordingly, Stat3 signalling is elevated in both psoriasis and IMQ-induced psoriasis-like lesions, according to several studies (Deng et al., 2016). In similar research, the authors indicated that paeoniflorin suppresses IMQ-induced psoriasis in mice via modulating Th17 expression levels and upregulation (Deng et al., 2016).

It was confirmed that IL-10 production offers therapeutic effects as typical anti-psoriatic therapy on numerous cell types. In this regard, the current study's findings reveal that treatment with all dosages of noscapine except 5 mg/kg increases the level of IL-10. Furthermore, the present study's results demonstrated the efficacy of high doses of noscapine in regulating the expression of proinflammatory cytokines. Although the precise processes and signalling pathways involved in lowering these cytokines are unknown, we suspect that the antiproliferative and anti-inflammatory impact of noscapine works by suppressing TNF-α /IFN-γ, resulting in more severe inflammation and keratinocyte proliferation. These findings support recent research that found the Wannachawee recipe dramatically suppressed TNF- and IFN-stimulated IL-17A, IL-22, and IL-23 release in HaCaT cells (Na Takuathung et al., 2018). Although precise anti-inflammatory signalling pathways of noscapine in psoriasis induction are still unknown, we hypothesise that noscapine's antiproliferative and anti-inflammatory activity occurs via TNF-α /IFN-γ reduction. Despite these findings, it is well documented that noscapine can be used as a treatment medication for psoriasis and has been extensively studied in clinical trials. The graphical abstract in Figure 8 indicates that noscapine reduces IMQ-induced psoriasis-like skin lesions.

## Conflicts of interest

The authors have declared that there is no conflict of interest.
